# Analysis of Potential Subtypes of SAPHO Syndrome

**DOI:** 10.3390/jcm14228116

**Published:** 2025-11-17

**Authors:** Hongji Duan, Wangna Tang, Xiaoli Deng

**Affiliations:** Beijing Jishuitan Hospital, Capital Medical University, Beijing 100035, China; bobduan300@163.com (H.D.); tangwangna@tmu.edu.cn (W.T.)

**Keywords:** SAPHO syndrome, subtypes, bone scan

## Abstract

**Objectives**: The purpose of this study is to identify clinical subtypes of SAPHO syndrome using cluster analysis, and to systematically investigate the associated clinical characteristics, therapeutic approaches, and short-term prognostic outcomes in order to enhance patient management. **Methods**: We recruited patients who had been diagnosed with SAPHO syndrome at Beijing Jishuitan Hospital. Bone lesions were assessed using a ^99^Tc bone scan. Based on bone lesions and clinical features, patients were categorized using a hierarchical clustering algorithm. Laboratory test results and prognostic differences were compared among clusters. **Results**: Overall, 135 patients were included. Cluster analysis identified three distinct clusters. Eighty-seven patients were assigned to cluster 1, characterized by anterior chest wall involvement; 74.7% also had skin involvement. Nineteen patients were assigned to cluster 2, characterized by spinal involvement, and 10.5% showed skin manifestations. Twenty-nine patients were assigned to cluster 3, characterized by peripheral bone involvement, with 24.1% exhibiting skin manifestations. Patients in cluster 3 were younger at disease onset (36.92 ± 16.62 years); their BASDAI and BASFI scores were lower (2.51 ± 1.18 and 0.89 ± 1.37, respectively). In cluster 1, significant reductions in Visual Analog Scale scores were observed at 1 and 6 months after treatment compared with baseline (7.08 vs. 2.59, *p* < 0.001, *n* = 63; 7.09 vs. 1.93, *p* < 0.001, *n* = 45). Similar improvements were noted in clusters 2 and 3 (7.18 vs. 3.00, *p* < 0.001, *n* = 11; 7.00 vs. 3.00, *p* < 0.001, *n* = 7; 6.70 vs. 2.65, *p* < 0.001, *n* = 20; 6.60 vs. 2.47, *p* < 0.001, *n* = 15). **Conclusions**: SAPHO syndrome may be classified into three subtypes: typical, axial, and peripheral. All subtypes show rapid improvement with timely treatment. Defining the clinical characteristics of these three subtypes can aid diagnosis and provide pathogenesis insights regarding this heterogeneous syndrome.

## 1. Introduction

SAPHO syndrome is a rare inflammatory disorder characterized by synovitis, acne, pustulosis, hyperostosis, and osteitis, from which its acronym is derived. The syndrome was first described in 1987 [1]. Diagnosis is primarily based on clinical manifestations because there currently is no universally accepted standard for the diagnosis, treatment, or prognosis of SAPHO syndrome. Commonly referenced diagnostic criteria include those proposed by Benhamou and colleagues in 1988 [2] and by Kahn in 1994, later revised in 2003 [3]. No specific clinical features or laboratory markers have been established as diagnostic indicators for SAPHO syndrome.

The clinical presentation of SAPHO syndrome is diverse, with variable effects on patients’ quality of life. Chronic nonbacterial osteomyelitis (CNO), its severe form chronic recurrent multifocal osteomyelitis (CRMO), and pustular arthro-osteitis (PAO) are considered closely related entities; they may represent limited forms of SAPHO syndrome [3,4,5,6]. Atypical manifestations and incomplete understanding of the disease can lead to substantial diagnostic delays [7,8]. Osteitis and hyperostosis—core features of SAPHO syndrome—usually involve multiple skeletal regions [9]. The most commonly affected site is the anterior chest wall (ACW), followed by the axial skeleton (including spine and sacroiliac joints) and the long bones of the limbs [10]. Most patients exhibit skin involvement, typically manifesting as palmoplantar pustulosis (PPP) or severe acne (SA) [9]. Over 70% of patients develop both skin and osteoarticular manifestations within 2 years of disease onset, although longer intervals have also been reported [11]. Many drugs have been used to manage SAPHO syndrome [12,13,14], but treatment responses considerably vary among patients.

These variations in clinical phenotype and therapeutic response complicate the diagnosis, treatment, and prognostic evaluation of SAPHO syndrome. This study aimed to classify the clinical characteristics of patients with SAPHO syndrome, identify distinct subtypes, and evaluate their associations with treatment response and prognosis. Such findings may contribute to more precise disease management. Cluster analysis offers an important approach to improving understanding of SAPHO syndrome and may aid its clinical diagnosis.

## 2. Materials and Methods

### 2.1. Patients

All patients were recruited from Beijing Jishuitan Hospital. The inclusion criteria were as follows: (1) fulfillment of the diagnostic criteria for SAPHO syndrome proposed by Kahn, with minor modifications [3]; (2) completion of a ^99^Tc bone scan for the assessment of bone and joint lesions; and (3) confirmation of lesions by computed tomography and magnetic resonance imaging, as evaluated by the same rheumatologist and radiologist. Exclusion criteria were infectious osteitis, bone tumors, infectious PPP, and noninflammatory bone-condensing lesions. This study was approved by the Ethics Committee of Beijing Jishuitan Hospital (approval number: K2023-134-02). Written informed consent to participate was obtained from each patient.

### 2.2. Clinical Evaluation

**Figure 1:**
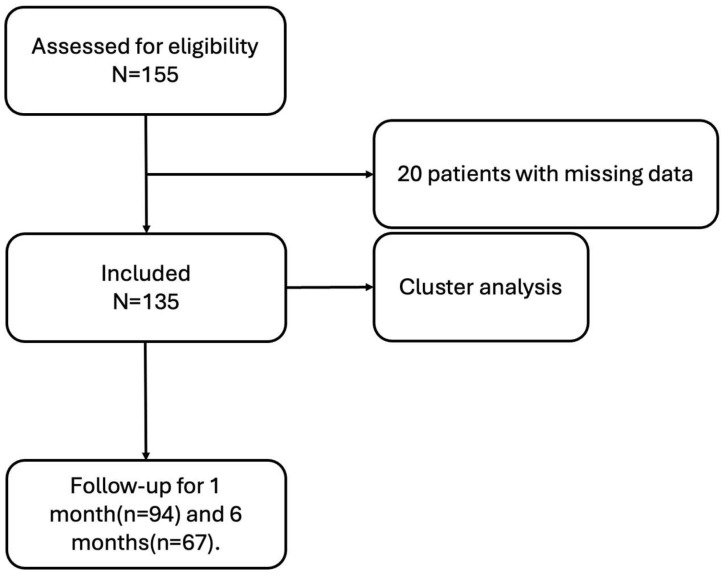
Flow diagram of the study.

The following clinical data were collected: sex, age, disease duration, bone and joint manifestations, skin manifestations, erythrocyte sedimentation rate (ESR), C-reactive protein (CRP), cytokines, bone metabolism markers, imaging findings, and pathological results (bone biopsy performed when deemed necessary by the clinician and the patient). Disease activity was assessed using the Visual Analog Scale (VAS), Bath Ankylosing Spondylitis Disease Activity Index (BASDAI), Bath Ankylosing Spondylitis Functional Index (BASFI), and Health Assessment Questionnaire (HAQ). All patients received treatment with iguratimod and alendronate. Follow-up evaluations were conducted at 1 month and 6 months after treatment (Figure 1).

### 2.3. Statistical Analysis

All data analyses were performed using R software (version 4.5.0). Categorical variables were analyzed using a hierarchical clustering algorithm with the Average Linkage method and Gower’s similarity coefficient. Variables included in the cluster analysis comprised the most commonly affected bone and joint locations, lesion distribution, bone lesion type, and skin involvement.

Group comparisons were performed as follows: normality was assessed using the Kolmogorov–Smirnov test. Descriptive data were expressed as mean (range) or number (%). The χ^2^ test or Fisher’s exact test was used for categorical variables. Normally distributed continuous variables were tested using the t-test. Non-normally distributed continuous variables were tested using the Mann-Whitney U test. Paired t-tests assessed changes in individual parameters across time points. All statistical tests were two-tailed, and *p*-values < 0.05 were considered statistically significant.

## 3. Results

### 3.1. Demographic and Clinical Characteristics

In total, 135 patients (95 women and 40 men) were included in this study, with a mean age of 44.49 ± 13.89 years. Bone lesions were predominantly symmetrical (85/135, 63.0%), and most presented as osteosclerosis (102/135, 75.6%). All patients exhibited osteoarticular symptoms. The frequencies of ACW, spinal, and peripheral bone or joint involvement were 65.2% (88/135), 40.7% (55/135), and 27.4% (37/135), respectively. Concurrent skin manifestations were present in 54.8% (74/135) of patients. The most common skin presentations were PPP in 28.1% (38/135), SA in 8.1% (11/135), and psoriasis vulgaris (PV) in 3.7% (5/135). Elevated ESR was observed in 41.5% (56/108), and elevated CRP in 24.4% (33/106). Bone biopsy was performed in 21 patients, and low-virulence bacteria were identified in 10 cases through bacterial culture or next-generation sequencing. The detailed demographic and clinical characteristics are summarized in Table 1.

### 3.2. Cluster Analysis

**Figure 2:**
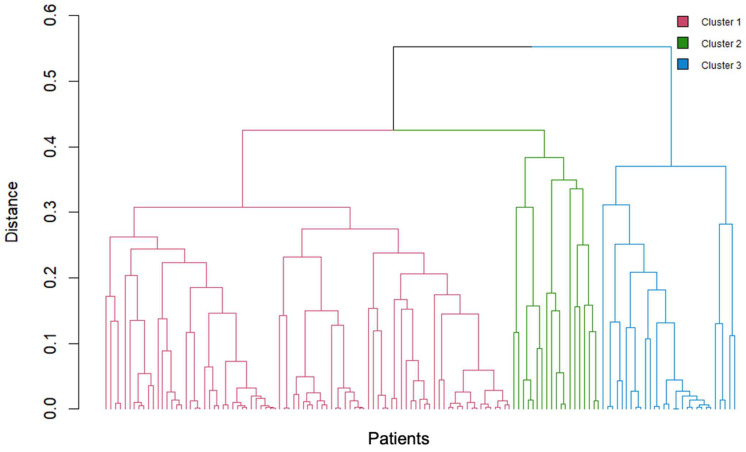
Three clusters of patients with SAPHO syndrome identified by cluster analysis. Colors indicate cluster membership.

Cluster analysis classified patients with SAPHO syndrome into three distinct clusters based on the distribution of bone and joint involvement, lesion type, and presence of skin manifestations. Each cluster demonstrated unique clinical characteristics (Figure 2).

**Figure 3:**
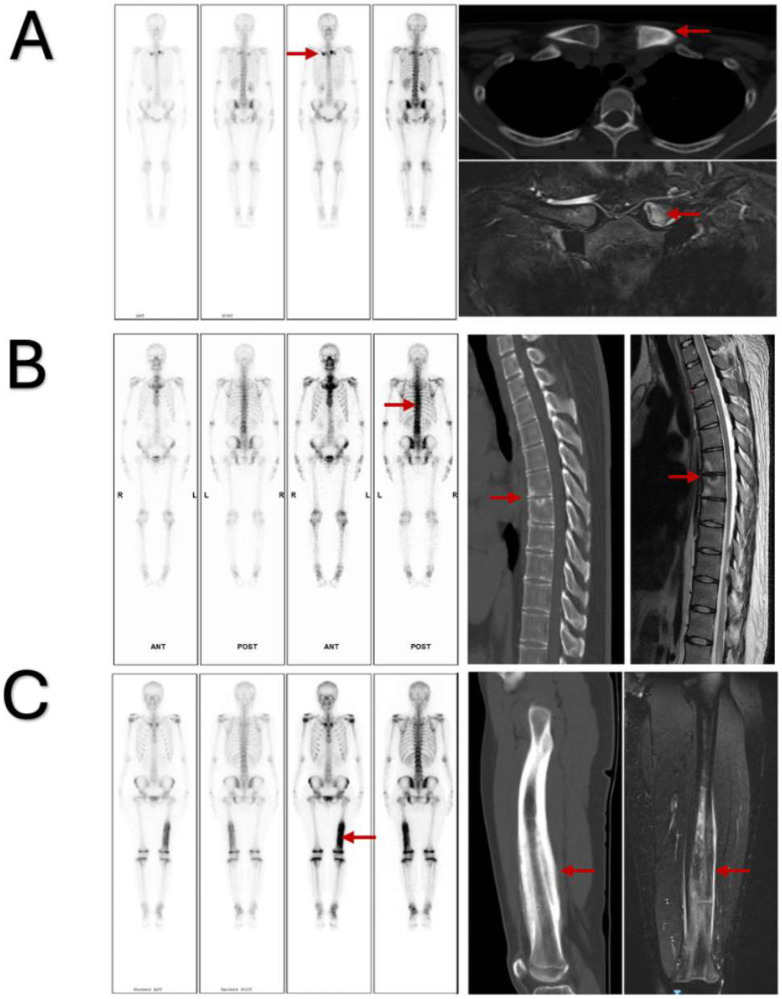
Representative whole-body bone scintigraphy, computed tomography, and magnetic resonance imaging findings in patients with SAPHO syndrome. (**A**): Cluster 1 (typical SAPHO syndrome); (**B**): Cluster 2 (axial SAPHO syndrome); (**C**): Cluster 3 (peripheral SAPHO syndrome).

Cluster 1, characterized by ACW and skin involvement, included 87 patients (Figure 3). The disease in this cluster could be defined as typical SAPHO syndrome. Bone lesions were primarily located in the ACW (88.5%) and spine (51.7%); osteosclerosis was the predominant feature (80.5%). Lesions were usually symmetrical (88.5%), and most patients presented with skin involvement (74.7%). This cluster represents the disease form most easily recognized in clinical practice.

Cluster 2, characterized by spinal involvement, included 19 patients (Figure 3). The disease in this cluster could be defined as axial SAPHO syndrome. Bone lesions were mainly located in the spine, without prominent osteosclerosis or bone destruction. Lesions tended to be asymmetrical, and skin manifestations were infrequent (10.5%).

Cluster 3, characterized by peripheral bone and joint involvement, included 29 patients (Figure 3). The disease in this cluster could be defined as peripheral SAPHO syndrome. Lesions were mainly found in peripheral bones and joints (75.9%); they predominantly exhibited osteosclerosis (96.6%). Most lesions involved a single site (86.2%), and skin manifestations were relatively uncommon (24.1%). This cluster represents the disease form most challenging to diagnose in clinical settings.

Patients in cluster 3 were younger at disease onset (36.92 ± 16.62 years). BASDAI and BASFI scores were lower in cluster 3 (2.51 ± 1.18 and 0.89 ± 1.37, respectively). Among the clusters, cluster 3 showed the highest proportion of osteosclerosis (96.6%). No significant differences were evident in ESR, CRP, cytokines, or bone metabolism markers among the clusters. Detailed comparisons are presented in Table 2.

**Figure 4:**
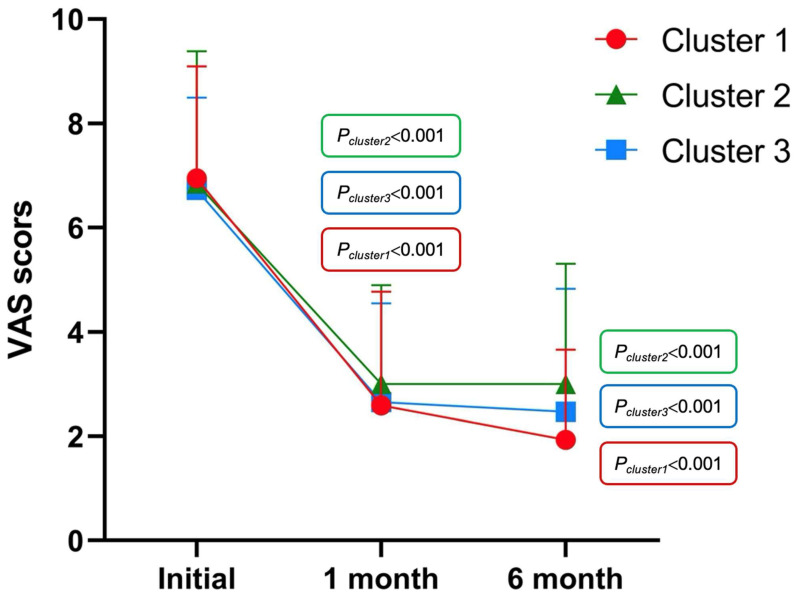
Improvement in VAS scores across the three clusters after treatment.

In cluster 1, significant reductions in VAS scores were observed at 1 and 6 months after treatment compared with baseline (7.08 vs. 2.59, *p* < 0.001, *n* = 63; 7.09 vs. 1.93, *p* < 0.001, *n* = 45). Similar improvements were noted in Clusters 2 and 3 (7.18 vs. 3.00, *p* < 0.001, *n* = 11; 7.00 vs. 3.00, *p* < 0.001, *n* = 7; 6.70 vs. 2.65, *p* < 0.001, *n* = 20; 6.60 vs. 2.47, *p* < 0.001, *n* = 15) (Figure 4).

## 4. Discussion

In this study, new subtypes of SAPHO syndrome were identified through cluster analysis based on clinical manifestations and radiologic findings.

Previous research has identified ACW involvement in 65–90% of patients, a characteristic feature of SAPHO syndrome [15,16]. Typically affected structures include the sternoclavicular joint, sternocostal joint, and clavicular ligament. Axial involvement occurs in 32–52% of cases [15]. In the present study, the typical SAPHO syndrome group (cluster 1) showed similar findings: ACW and spinal involvement were observed in 88.5% and 51.7% of patients, respectively. On bone scintigraphy, increased uptake in the sternoclavicular region, known as the “bullhead sign,” is considered a characteristic imaging feature of SAPHO syndrome [17]. The progression of bone and joint changes in this area can be divided into three stages: soft tissue swelling, bone erosion, and new bone formation with hyperostosis and osteosclerosis [15]. In our study, cluster 1 mainly involved the ACW and spine; osteosclerosis was the predominant manifestation (80.5%). Therefore, bone scintigraphy is recommended for patients with ACW involvement to prevent missed diagnoses of concomitant spinal lesions. Skin manifestations are also common; characteristic skin changes may appear before the onset of arthritis (25%), concurrently with joint symptoms (63.5%), or after arthritis develops (5.8%) [18]. In our study, most patients in cluster 1 exhibited skin involvement (74.7%). Therefore, this subtype is more readily recognized by rheumatologists in clinical settings. Patients in the typical SAPHO syndrome group (cluster 1) generally had longer disease duration, with more severe pain and functional impairment. However, follow-up results showed substantial symptom relief within 1 month after treatment initiation, with sustained improvement for at least 6 months. At present, it remains uncertain whether early aggressive treatment is warranted for this subtype.

Axial skeleton involvement may occur in several forms and combinations, including sclerosis of one or more vertebral bodies with osteosclerosis, osteolytic lesions with varying degrees of vertebral collapse, and spondylitis with or without discitis [15]. In the present study, patients with axial SAPHO syndrome (cluster 2) exhibited bone lesions characterized by osteosclerosis and bone destruction. Lesions were typically multifocal (94.7%), and only a small proportion of patients presented with skin manifestations (10.5%). Axial SAPHO syndrome (cluster 2) should be distinguished from axial spondyloarthritis (axSpA) in clinical practice. Compared with the findings in axSpA, magnetic resonance imaging findings in SAPHO syndrome more commonly show bone marrow edema at the anterior corners of vertebrae, bone erosion, and swelling of the intervertebral disks and endplates [19]. In axSpA, elevated levels of ESR and tumor necrosis factor (TNF) are frequently observed; however, we found that ESR was elevated in some patients with axial SAPHO syndrome (66.7%), but TNF was not increased. Previous studies have shown that patients with spinal involvement often experience more severe clinical manifestations [20]. Similarly, we noted that axial SAPHO syndrome (cluster 2) had a greater impact on spinal function and overall quality of life. Nonetheless, symptoms in most patients improved rapidly after treatment.

Long bone involvement is uncommon in adults; such sites are frequently affected in children, typically involving the distal femur and proximal or distal tibia. Single-bone involvement has also been reported, and the mandible may be affected, revealed diffuse unilateral sclerosis [21]. In the present study, peripheral SAPHO syndrome (cluster 3) was mainly characterized by peripheral bone and joint involvement. Affected patients often lacked typical skin manifestations and experienced frequent diagnostic delays. Previous cohort studies have shown diagnostic delays of up to 9 years in some SAPHO cases, resulting in irreversible structural damage, increased disease burden, and disability [8,22]. Therefore, improvements to diagnostic accuracy for peripheral SAPHO syndrome (cluster 3) are particularly important. Patients in this cluster presented at a younger age, had a shorter disease duration, and predominantly exhibited osteosclerosis as the primary bone lesion. Pain intensity and functional impairment were milder than in other clusters. Bone biopsy is often required in these patients to exclude alternative diagnoses.

At present, there is no standardized treatment protocol for SAPHO syndrome worldwide, and its exact pathogenesis remains unclear. Several cytokines are believed to contribute to disease development. Previous studies have shown that proinflammatory cytokines—such as TNF-α, interleukin (IL)-1β, IL-6, and IL-8—are strongly expressed in patients with SAPHO syndrome, promoting an amplified inflammatory response. These cytokines facilitate disease progression by enhancing inflammation, inducing acute-phase reactions, and promoting chemotaxis. In contrast, the anti-inflammatory cytokine IL-10 and transforming growth factor-β exert protective effects by regulating immune responses and suppressing excessive inflammation [23]. A systematic review suggested that TNF inhibitors are the preferred treatment for patients who do not respond to conventional therapy. For patients who show insufficient response to TNF blockade, IL-1 inhibitors and biologic agents targeting the IL-17/IL-23 axis may be considered [12]. Moreover, the interaction among the transcription factor forkhead box O1 (FoxO1), *Propionibacterium acnes*, NLR family pyrin domain-containing protein 3 (NLRP3) inflammasomes, and IL-1β may contribute to the pathogenesis of osteitis [24]. Currently, no single therapeutic regimen can address all manifestations of SAPHO syndrome [14]. In the present study, the therapeutic responses of patients across the three identified clusters were followed for a short duration. Treatment decisions were made jointly by physicians and patients without external intervention. All three clusters showed rapid symptomatic improvement after treatment. However, the potential for different subtypes to require distinct therapeutic strategies warrants further investigation.

This study had some limitations. The sample size was relatively small, and the follow-up period was short. Future studies should include larger cohorts and longer observation periods. Further research is also needed to elucidate pathogenic mechanisms underlying differences among the subtypes.

## 5. Conclusions

This study is the first to use cluster analysis to classify SAPHO syndrome based on clinical characteristics. The results suggest that SAPHO syndrome can be divided into three subtypes: typical, axial, and peripheral. All subtypes demonstrated rapid improvement after timely treatment. Thus, early and accurate diagnosis is particularly important for optimal management.

## Figures and Tables

**Table 1:** Baseline demographic and clinical characteristics of patients with SAPHO syndrome.

Demographic and Clinical Characteristics	
Demographic characteristics	
Sex, female/male	95/40
Age, mean (S.D.), years	44.49 (13.89)
Disease duration, mean (S.D.), months	59.10 (81.50)
Smoking, *n* (%)	26 (19.3%)
Alcohol intake, *n* (%)	30 (22.2%)
Clinical characteristics	
Osteoarticular symptoms	
Single site involvement, *n* (%)	26 (19.3%)
Symmetry of lesion site, *n* (%)	85 (63.0%)
Bone destruction, *n* (%)	12 (8.9%)
Osteosclerosis, *n* (%)	102 (75.6%)
Anterior chest wall, *n* (%)	88 (65.2%)
Spine, *n* (%)	55 (40.7%)
Peripheral bones and joints, *n* (%)	37 (27.4%)
Skin manifestations	
PPP, *n* (%)	38 (28.1%)
PV, *n* (%)	5 (3.7%)
SA, *n* (%)	11 (8.1%)
Nonspecific rash, *n* (%)	22 (16.7%)
None	61 (45.2%)
Disease assessment index	
VAS, mean (S.D.)	6.88 (2.12)
BASDAI, mean (S.D.)	3.46 (1.92)
BASFI, mean (S.D.)	1.79 (2.02)
HAQ, mean (S.D.)	0.41 (0.48)
Laboratory tests	
Elevated ESR (*n* = 108), n (%)	56 (41.5%)
Elevated CRP (*n* = 106), n (%)	33 (24.4%)
TNF-α (*n* = 81), mean (S.D.)	2.50 (1.41)
IL-6 (*n* = 82), mean (S.D.)	8.82 (7.57)
IL-8 (*n* = 82), mean (S.D.)	10.10 (12.07)
tP1NP (*n* = 71), mean (S.D.)	74.28 (97.57)
β-CTX (*n* = 36), mean (S.D.)	4.69 (11.91)
PTH (*n* = 99), mean (S.D.)	35.31 (13.11)
25-(OH)VD3 (*n* = 108), mean (S.D.)	22.88 (9.33)
Pathogenic bacteria (*n* = 21), *n* (%)	10 (47.6%)

PPP, palmoplantar pustulosis; PV, psoriasis vulgaris; SA, severe acne; VAS, Visual Analog Scale; BASDAI, Bath Ankylosing Spondylitis Disease Activity Index; BASFI, Bath Ankylosing Spondylitis Functional Index; HAQ, Health Assessment Questionnaire.

**Table 2:** Comparison of clinical and laboratory features among clusters.

Demographic Characteristics	Cluster 1 (*n* = 87)	Cluster 2 (*n* = 19)	Cluster 3 (*n* = 29)	*p* Values *	*p* Values **	*p* Values ***
Sex, female	64 (73.6%)	11 (57.9%)	20 (69.0%)	0.264	0.638	0.541
Age	46.75 (12.83)	45.68 (10.09)	36.92 (16.62)	0.547	0.003	0.047
Disease duration	60.68 (76.77)	54.90 (70.45)	57.14 (102.34)	0.401	0.012	0.374
Smoking	18 (20.7%)	4 (21.1%)	4 (13.8%)	0.972	0.586	0.509
Alcohol intake	23 (26.4%)	6 (31.6%)	1 (3.4%)	0.777	0.007	0.007
Osteoarticular symptoms						
Single site involvement	0	1 (5.3%)	25 (86.2%)	0.032	<0.001	<0.001
Symmetry of lesion site	77 (88.5%)	7 (36.6%)	1 (3.4%)	<0.001	<0.001	0.002
Bone destruction	6 (6.9%)	1 (5.3%)	5 (17.2%)	0.795	0.100	0.220
osteosclerosis	70 (80.5%)	4 (21.1%)	28 (96.6%)	<0.001	0.038	<0.001
Anterior chest wall	77 (88.5%)	7 (36.8%)	4 (13.8%)	<0.001	<0.001	0.063
Spine	45 (51.7%)	9 (47.4%)	1 (3.4%)	0.803	<0.001	<0.001
Peripheral bones and joints	12 (13.8%)	3 (15.8%)	22 (75.9%)	0.821	<0.001	<0.001
Skin manifestations	65 (74.7%)	2 (10.5%)	7 (24.1%)	<0.001	<0.001	0.237
VAS	6.94 (2.15)	6.84 (2.54)	6.72 (1.77)	0.950	0.457	0.482
BASDAI	3.79 (2.09)	3.42 (1.57)	2.51 (1.18)	0.578	0.003	0.033
BASFI	1.99 (2.12)	2.26 (2.01)	0.89 (1.37)	0.396	0.001	0.002
HAQ	0.42 (0.51)	0.57 (0.46)	0.25 (0.33)	0.103	0.060	0.008
Laboratory tests						
Elevated ESR (*n* = 92)	33 (50.0%)	10 (66.7%)	13 (48.1%)	0.269	1.000	0.337
Elevated CRP (*n* = 90)	20 (30.8%)	4 (30.8%)	9 (32.1%)	1.000	1.000	0.930

*: cluster 1 vs. cluster 2; **: cluster 1 vs. cluster 3; ***: cluster 2 vs. cluster 3.

## Data Availability

The original data supporting the findings of this study are included in the article. Further inquiries can be directed to the corresponding author.

## Abbreviations

The following abbreviations are used in this manuscript:

SAPHO	synovitis, acne, pustulosis, hyperostosis, and osteitis
CRMO	chronic recurrent multifocal osteomyelitis
CNO	chronic nonbacterial osteomyelitis
PAO	pustular osteoarthritis
ACW	anterior chest wall
PPP	palmoplantar pustulosis
SA	severe acne
ESR	erythrocyte sedimentation rate
CRP	C-reactive protein
VAS	Visual Analog Scale
BASDAI	Bath Ankylosing Spondylitis Disease Activity Index
BASFI	Bath Ankylosing Spondylitis Functional Index
HAQ	Health Assessment Questionnaire
PV	psoriasis vulgaris
axSpA	axial spondyloarthritis
